# Co-transduction of Apaf-1 and caspase-9 highly enhances p53-mediated apoptosis in gliomas

**DOI:** 10.1038/sj.bjc.6600061

**Published:** 2002-02-12

**Authors:** N Shinoura, S Sakurai, F Shibasaki, A Asai, T Kirino, H Hamada

**Affiliations:** Department of Molecular Biotherapy Research, Cancer Chemotherapy Center, Cancer Institute, 1-37-1 Kami-Ikebukuro, Toshima-ku, Tokyo 170-8455, Japan; Department of Neurosurgery, Tokyo University, 7-3-1 Hongo, Bunkyo-ku, Tokyo 113-8655, Japan; Department of Neurosurgery, Komagome Metropolitan Hospital, 3-18-22 Hon-Komagome, Bunkyo-ku, Tokyo 113-8677, Japan; Department of Microbiology, Komagome Metropolitan Hospital, 3-18-22 Hon-Komagome, Bunkyo-ku, Tokyo 113-8677, Japan; Department of Biochemistry, Kyoritsu College of Pharmacy, Shibakoen 1-5-30, Minato-ku, Tokyo 105-8512, Japan; CREST (Core Research for Evolutional Science and Technology), 2-6-15 Shibakoen, Minato-ku, Tokyo, 105-0011, Japan; Department of Molecular Medicine, Sapporo Medical University, Sapporo 060-8556, Japan

**Keywords:** apoptosis, p53, Apaf-1, caspase-9, glioma

## Abstract

Mutation of the p53 gene plays a critical role in the development of cancer and response to cancer therapy. To analyze the mechanism of cancer development and to improve cancer therapy, it is important to assess which genes are downstream components of p53 in cancers, and whether the expression levels of these genes affect p53-mediated apoptosis. In this study, we transduced the wild type p53 gene along with the Apaf-1 and caspase-9 genes via adenovirus vectors into U251 and U-373MG glioma cells harbouring a mutated p53, and evaluated the degree of apoptosis. Co-induction of Apaf-1 and caspase-9 genes highly enhanced p53-mediated apoptosis in glioma cells. Induction of wild type p53 enhanced the expression levels of Bax, p21/WAF1, and Fas protein. To determine which gene is activated by wild type p53 induction and, in turn, activates Apaf-1 and caspase-9, we transduced the Bax, p21/WAF1 or Fas gene via adenovirus vector to U251 cells to achieve a similar expression level as that induced by the Adv for p53 in U251 cells. U251 cells transduced with Fas concomitant with the Apaf-1 and caspase-9 genes underwent drastic apoptosis. This suggests that induction of wild type p53 upregulates Fas, which in turn may play a role in the activation of Apaf-1 and caspase-9. These results are important for analyzing the mechanism of tumour development and for predicting the therapeutic effect of p53 replacement gene therapy in a particular patient.

*British Journal of Cancer* (2002) **86**, 587–595. DOI: 10.1038/sj/bjc/6600061 www.bjcancer.com

© 2002 Cancer Research UK

## 

The p53 gene is one of the most frequently mutated genes in cancers, and inactivation of the p53 gene is critical in the development and malignant progression of cancers. Genes that have been reported to be involved in p53-mediated apoptosis include Bax (Miyashita and Reed, 1995), Fas (Owen-Schaub *et al*, 1995) and p53-inducible genes known as PIGs (Polyak *et al*, 1997). Recently, Apaf-1 and caspase-9 were found to be essential downstream components of p53 in Myc-induced apoptosis in early-passage mouse embryonal fibroblasts (Soengas *et al*, 1999). Therefore, it would be interesting to know whether Apaf-1 and caspase-9 are also downstream components of p53-mediated apoptosis in cancers.

To restore p53 function in cancers with mutated p53, p53 gene replacement therapy using viral vectors has been performed. Transfection of wild type p53 in *in vitro* and *in vivo* animal experiments has been shown to induce apoptosis of various cancers (for review, see Harris (1996), as well as in a clinical study (Roth *et al*, 1996)). Roth *et al* (1996) reported that in six of nine patients with lung cancer who underwent retrovirus-mediated transfer of the p53 gene, tumour regression or growth stabilization occurred without vector-related toxic effects. However, some tumours are resistant to p53 gene therapy. To be able to predict the effect of p53 gene therapy in a particular patient, it would be important to evaluate which genes are closely involved in p53-mediated apoptosis in cancers. Moreover, transduction of those genes into cancers may be beneficial in augmenting the effect of p53 gene therapy. In this study, we evaluated whether induction of the Apaf-1 and/or caspase-9 genes increases the degree of p53-mediated apoptosis, and found that co-induction of Apaf-1 and caspase-9 genes highly augments p53-mediated apoptosis in gliomas.

## MATERIALS AND METHODS

### Cell lines

The U-373MG glioma cell line was obtained from American Type Culture Collection (ATCC, Manassas, VA, USA). The U251 glioma line was obtained from the Tumor Registry at the Division of Cancer Treatment, National Cancer Institute (Frederick, MD, USA). Each cell line was maintained as described previously (Shinoura *et al*, 1998).

### Generation of the adenoviral vectors

The *Xho*I/*Pst*I fragment from pSXVtat-hp21 containing the full-length human p21/WAF1 cDNA, which was kindly provided by Dr Koh (Massachusetts General Hospital Cancer Center, Charlestown, MA, USA), was inserted into the *Xho*I/*Pst*I site of pBluescriptSK+ (Stratagene, La Jolla, CA, USA), which generated pSK+hp21. The *Xho*I/*Bam*HI site of pSK+hp21 was inserted into the *Xho*I/*Bgl*II site of pCAcc (Yoshida and Hamada, 1997), which generated pCA-hp21. The cosmid pAxCA-hp21 was generated by inserting the *Sal*I/*Hind*III expression cassette (blunt end) from pCA-hp21 into the *Swa*I site of the cosmid pAxcw.

Adenovirus (Adv) for Apaf-1 (Adv-APAF1), the Adv for caspase-9 (Adv-Casp9), Adv for Bax (Adv-Bax), Adv for Fas (Adv-Fas), and the Adv for p53 (Adv-p53) were generated and transduced to cells as described previously (Takayama *et al*, 1998; Shinoura *et al*, 1999a, 2000a,b). The total multiplicities of infection (MOIs) of adenovirus used to infect each cell preparation was kept the same in all experiments by supplementing with the Adv with deleted E1 and E3 (Adv-dE) (Kanegae *et al*, 1995).

### Assessment of cell death

The degree of cell death was assessed by determining the percentage of cells that had died, and the degree of DNA fragmentation, as described previously (Shinoura *et al*, 1999a).

Electron microscopic analysis for apoptotic cell death was performed as described previously (Shinoura *et al*, 1998).

### Detection of Fas

FACS analysis of Fas expression on the cell surface was performed as described previously (Shinoura *et al*, 1998).

### Immunoblot analysis

Immunoblot analysis was performed using the ECL kit (Amersham, Buckinghamshire, UK), as previously described (Shinoura *et al*, 1998). The primary antibodies we used were: mouse anti-human caspase-3 antibody (Transduction Laboratories, #C31720, Lexington, KY, USA), mouse anti-poly(ADP-ribose) polymerase (PARP) monoclonal antibody (BIOMOL Research Laboratories, USA-250, Plymouth Meeting, PA, USA), mouse anti-human p53 monoclonal antibody (Oncogene Science, #OP09, Cambridge, MA, USA), mouse anti-human Bax monoclonal antibody (Medical and Biological Laboratories, #M010-3, Nagoya, Japan), rabbit anti-human Bcl-x polyclonal antibody (Transduction Laboratories, #B22630, Lexington, KY, USA), rabbit anti-human caspase-9 polyclonal antibody (IMGENEX, #IMG-123, San Diego, CA, USA), rabbit anti-Apaf-1 polyclonal antibody (ProSci, #2013, Poway, CA, USA), mouse anti-human p21 monoclonal antibody (Pharmingen, #15091A), and mouse anti-β-actin monoclonal antibody (Sigma, #A-5441, St. Louis, MO, USA). The secondary antibodies we used were: horseradish peroxidase-conjugated rabbit anti-mouse IgG+A+M (H+L) (Zymed Laboratories, #61-6420, San Francisco, CA, USA) for caspase-3, PARP, p53, Bax, p21 and β-actin, or horseradish peroxidase-conjugated donkey anti-rabbit IgG [F(ab′)_2_] (Amersham, #NA9340) for Apaf-1, caspase-9 and Bcl-X_L_.

## RESULTS

### Co-transduction of Apaf-1 and caspase-9 highly enhanced p53-mediated apoptosis in gliomas

**Figure 1:**
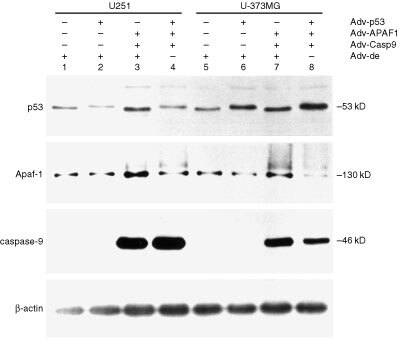
Immunoblot analysis of p53, Apaf-1, caspase-9 and β-actin protein extracted from U251 and U-373MG cells 48 h after being infected with Adv-p53, Adv-APAF1, Adv-Casp9, Adv for NCre (Adv-Cre) or Adv-dE in the indicated combinations. The MOI of Adv used to infect each glioma cell preparation is noted in parentheses. The total MOI was kept constant by supplementing with Adv-dE. Lane 1, U251 cells infected with Adv-dE (400); Lane 2, U251 cells co-infected with Adv-p53 (100) and Adv-dE (300); Lane 3, U251 cells co-infected with Adv-APAF1 (100), Adv-Casp9 (100), Adv-Cre (100) and Adv-dE (100); Lane 4, U251 cells co-infected with Adv-p53 (100), Adv-APAF-1 (100), Adv-Casp9 (100) and Adv-Cre (100); Lane 5, U-373MG cells infected with Adv-dE (400); Lane 6, U-373MG cells co-infected with Adv-p53 (100) and Adv-dE (300); Lane 7, U-373MG cells co-infected with Adv-APAF1 (100), Adv-Casp9 (100), Adv-Cre (100) and Adv-dE (100); Lane 8, U-373MG cells co-infected with Adv-p53 (100), Adv-APAF-1 (100), Adv-Casp9 (100) and Adv-Cre (100).

To investigate whether transduction of Apaf-1 or caspase-9 augments p53-mediated apoptosis, we selected the U251 and U-373MG glioma cell lines which show a similar level of susceptibility for Adv infection (Shinoura *et al*, 2000a). Transfection of Adv-p53 into U251 cells, as well as into U-373MG cells, induced the expression of exogenous wild type p53 ( Figure 1

, lanes 2, 4, 6, 8), the size of which is larger than that of endogenous mutated p53 (Figure 1, lanes 1, 3, 5, 7). Transfection of Adv-Casp9 induced the expression of caspase-9 (Figure 1, lanes 3, 4, 7, 8). Transfection of Adv-APAF1 into these two cell lines enhanced the expression of Apaf-1 (Figure 1, lanes 3, 7). As shown in Figure 1, the molecular weight (M_r_) of endogenous, mutant p53 in U251 cells (Figure 1, lanes 1, 3) and in U-373MG cells (Figure 1, lanes 5, 7) is smaller than that of wild type p53 in the respective cells (Figure 1, lanes 2, 4, 6, 8). It has been reported that U251 and U-373MG cells carry a point mutation in codon 273 of the p53 gene (Van Meir *et al*, 1994). The expression level of endogenous caspase-9 in U251 and U-373MG cells was much lower than that of exogenous caspase-9 induced by Adv-Casp9 infection.

**Figure 2:**
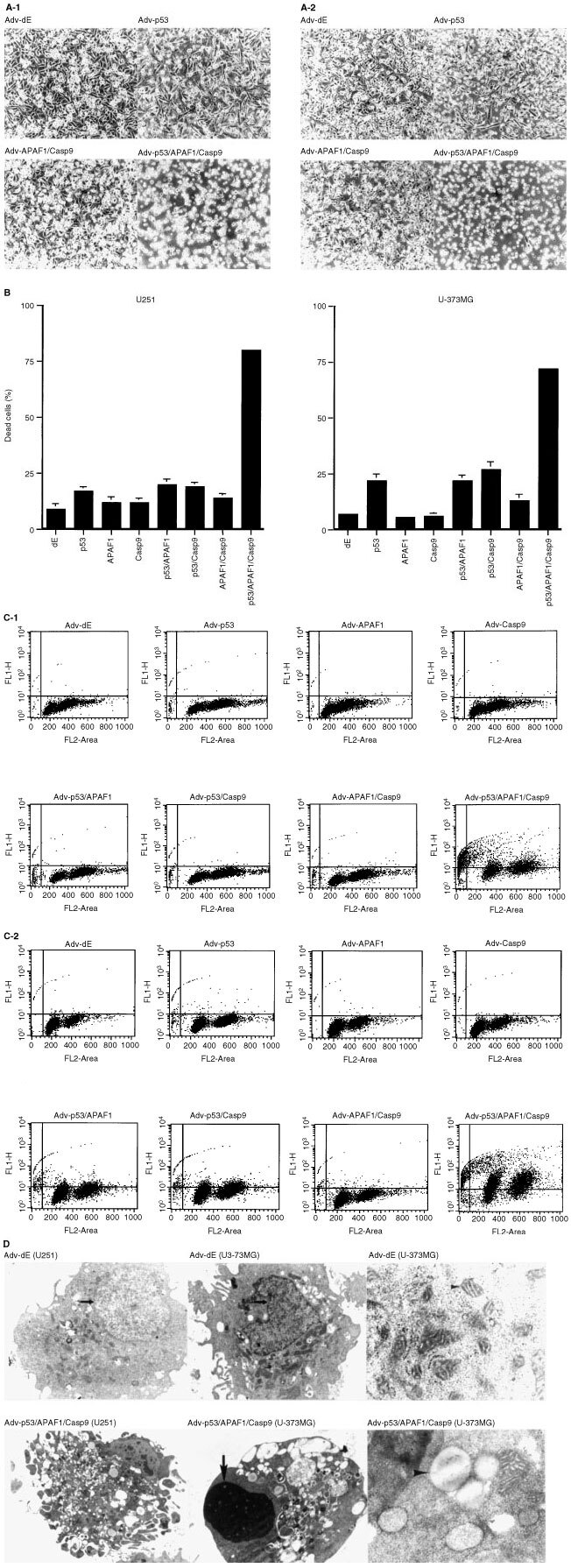
(**A**) Microscopic photographs of U251 (A-1) and U-373MG (A-2) cells that had been infected with Adv-p53 (MOI 100); Adv-APAF1 (MOI 100), and Adv-Casp9 (MOI 100); Adv-p53 (MOI 100), Adv-APAF1 (100), and Adv-Casp9 (100); or Adv-dE. The cells were examined 3 days after infection (original magnification ×100). The total MOI in all of the experiments was kept the same by supplementing with Adv-dE. (**B**) Percentage of cells that had died among U251 and U-373MG cells, measured by trypan blue exclusion 3 days after infection with Adv-p53 (MOI 100), Adv-APAF1 (MOI 100), Adv-Casp9 (MOI 100), or Adv-dE, or co-infection of these Advs. The total MOI was kept constant by supplementing with Adv-dE. The mean±standard deviation of the percentage of dead cells in three preparations of two independent experiments is shown. (**C**) DNA fragmentation of U251 (C-1) or U-373MG (C-2) cells infected with Adv-p53 (MOI 100), Adv-APAF1 (MOI 100), Adv-Casp9 (MOI 100), or Adv-dE, or co-infected with the indicated combinations of these Advs. The total MOI was kept constant by supplementing with Adv-dE. The assay was performed as described in the Materials and Methods 3 days after infection. The X-axis represents the propidium iodide-related fluorescence and the Y-axis represents the Br-dUTP-related fluorescence. The points in the upper left and upper right areas of each panel represent apoptotic cells with fragmented DNA. (**D**) Ultrastructural analysis of U251 and U-373MG cells after being co-infected with Adv-p53 (MOI 100), Adv-APAF1 (MOI 100) and Adv-Casp9 (MOI 100), or infected with Adv-dE. The total MOI was kept constant. Upper left panel: U251 cells 3 days after being infected with Adv-dE (×8000); upper middle panel: U-373MG cells 3 days after being infected with Adv-dE (×7000); upper right panel: U-373MG cells 1 day after being infected with Adv-dE (×40000); lower left panel: U251 cells 3 days after being co-infected with Adv-p53, Adv-APAF1 and Adv-Casp9 (×5000); lower middle panel: U-373MG cells 3 days after being co-infected with Adv-p53, Adv-APAF1 and Adv-Casp9 (×8000); lower right panel: U-373MG cells 1 day after being co-infected with Adv-p53, Adv-APAF1 and Adv-Casp9 (×8000). One day after being co-infected with Adv-p53, Adv-APAF1 and Adv-Casp9 (×40 000), most of the U-373MG cells showed mitochondrial damage (arrowhead in lower right panel) despite a normal nucleus, whereas 1 day after being infected with Adv-dE, U-373MG cells showed normal mitochondria (arrowhead in upper right panel) and nucleus. Apoptotic bodies (lower left panel) and condensation of chromatin (arrow in lower middle panel) were present in U251 and U-373MG cells 3 days after being co-infected with Adv-p53, Adv-APAF1 and Adv-Casp9.

We evaluated the effect of co-infecting Adv-p53, Adv-APAF1 and Adv-Casp9 into U251 and U-373MG cells on the degree of cell death and DNA fragmentation. The resultant percentage of cell death and degree of apoptosis, which was assessed by the degree of DNA fragmentation, in both cell lines were analyzed. U251 ( Figure 2A

-1) and U-373MG (Figure 2A-2) cells that had been co-infected with Adv-p53, Adv-APAF1 and Adv-Casp9, were effectively killed 3 days after infection. The percentage of cells that had died among U251 cells and among U-373MG cells co-infected with Adv-p53, Adv-APAF1 and Adv-Casp9 (80±1.3 and 72±1.3%, respectively) was much higher than the percentage of cells that had died among U251 cells and U-373MG cells infected with Adv-p53 alone (17±1.9 and 22±3.0%, respectively), or co-infected with Adv-APAF1 and Adv-Casp9 (14±1.9 and 13±2.9%, respectively) (Figure 2B).

The Br-dUTP uptake assay for the detection of DNA fragments revealed that in U251 cells, 49% of the cells co-infected with Adv-p53, Adv-APAF1 and Adv-Casp9 contained fragmented DNA, while less than 2% of the cells infected with one or two of Adv-p53, Adv-APAF1, and Adv-Casp9 contained fragmented DNA (Figure 2C-1). Although the degree of apoptosis in U-373MG cells infected with Adv-p53 was more remarkable than that in U251 cells infected with Adv-p53, the U-373MG cells showed a similar pattern (Figure 2C-2). The percentage of cells with fragmented DNA among U-373MG cells co-infected with Adv-p53, Adv-APAF1 and Adv-Casp9 was 70%, whereas that among U-373MG cells infected with one or two of Adv-p53, Adv-APAF1 and Adv-Casp9 was less than 3%, except among the cells co-infected with Adv-p53 and Adv-APAF1 (11%) or with Adv-p53 and Adv-Casp9 (14%) (Figure 2C-2).

These results indicate that co-infection of Adv-p53, Adv-APAF1 and Adv-Casp9 induced remarkably enhanced apoptotic cell death in comparison with that induced by single or double infection of Adv-p53, Adv-APAF1 or Adv-Casp9 in the two glioma cell lines.

Electron microscopic analysis of glioma cells co-infected with Adv-p53, Adv-APAF1 and Adv-Casp9, revealed apoptotic bodies (Figure 2D: lower left panel) and condensed chromatin in the nuclei (Figure 2D: lower middle panel) 3 days after infection. These are features of apoptotic cell death. Moreover, as early as day 1 after co-infection of Adv-p53, Adv-APAF1 and Adv-Casp9 in U-373MG cells, most of the mitochondria were damaged (Figure 2D: lower right panel), while infection of Adv-dE did not induce damage to the mitochondria (Figure 2D: upper right panel).

**Figure 3:**
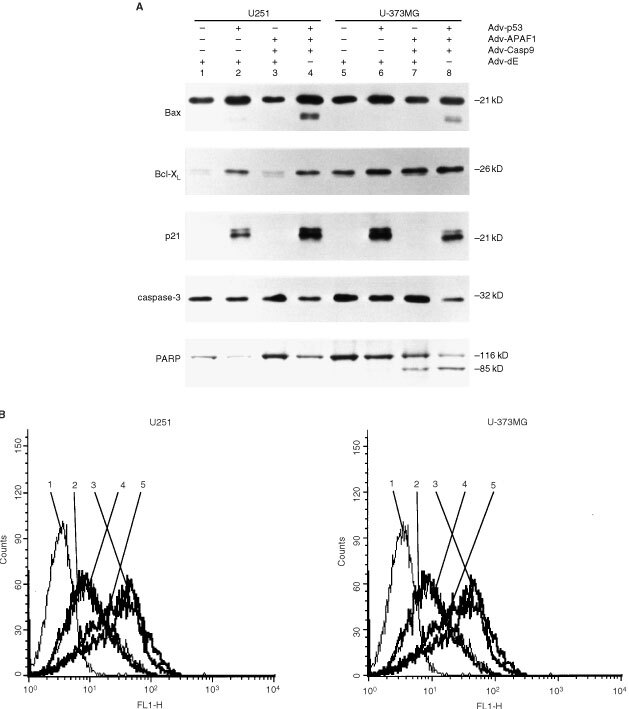
(**A**) Immunoblot analysis of Bax, Bcl-X_L_, p21, caspase-3, PARP and β-actin protein extracted from U251 and U-373MG cells 48 h after being infected with Adv-p53 (MOI 100), Adv-APAF1 (MOI 100), Adv-Casp9 (100) or Adv-dE, or co-infected with various combinations of these Advs. The MOI of each Adv used to infect the particular glioma cell preparation is the same as that in Figure 1. The control for equal protein loading in the lanes is the same as that in Figure 1. (**B**) Expression of Fas on the surface of U251 and U-373MG cells as measured by FACS after being infected with Adv-p53, Adv-APAF1, Adv-Casp9, or Adv-dE, or co-infected with various combinations of these Advs. U251 and U-373MG cells were stained with anti-Fas antibody as described in the Materials and Methods. The total MOI was kept constant by supplementing with Adv-dE. The data are presented as the log peak fluorescence intensity of each cell line infected with the indicated adenovirus(es) and stained with the indicated antibody: (1) isotype-matched control 2 days after being infected with Adv-dE (MOI 400); (2) anti-Fas antibody 2 days after being infected with Adv-dE (MOI 400); (3) anti-Fas antibody 2 days after being co-infected with Adv-p53 (MOI 100) and Adv-dE (MOI 300); (4) anti-Fas antibody 2 days after being co-infected with Adv-APAF1 (MOI 100), Adv-Casp9 (MOI 100), Adv-Cre (MOI 100) and Adv-dE (MOI 100); and (5) anti-Fas antibody 2 days after being co-infected with Adv-p53 (MOI 100), Adv-APAF1 (MOI 100), Adv-Casp9 (MOI 100), and Adv-Cre (MOI 100).

To evaluate the mechanism of apoptosis induced by co-infection with Adv-p53, Adv-APAF1 and Adv-Casp9 in glioma cells, we examined the expression of apoptosis-related genes including Bax, p21/WAF1, Bcl-X_L_, caspase-3, PARP and Fas. It was found that p53 gene transfection increased the expression levels of Bax and p21/WAF1 in U251 and U-373MG cells ( Figure 3A

, lanes 2, 4, 6 and 8), and Bcl-X_L_ in U251 cells (Figure 3A, lanes 2 and 4). In both cells co-infected with Adv-p53, Adv-APAF1 and Adv-Casp9 (Figure 3A, lanes 4 and 8), anti-Bax antibody recognized a 21-kDa protein and an 18-kDa protein, the latter of which has been reported to play a role in the regulation of apoptosis (Oltvai *et al*, 1993; Thomas *et al*, 1996). Induction of p53 alone and induction of Adv-p53, Adv-APAF1 and Adv-Casp9 induce markedly increased and similar expression of Fas (Figure 3B). In U-373MG cells, the expression level of caspase-3 after co-infection of Adv-p53, Adv-APAF1 and Adv-Casp9 (Figure 3A, lane 8) was reduced in comparison with that after infection of Adv-p53 or co-infection of Adv-APAF1 and Adv-Casp9. This suggests that some of the caspase-3 molecules in the U-373MG cells co-infected with Adv-p53, Adv-APAF1 and Adv-Casp9, were cleaved, although the antibody used in this study did not recognize the cleaved product of caspase-3. In accordance with this result, the majority of the PARP molecules in the U-373MG cells co-infected with Adv-p53, Adv-APAF1 and Adv-Casp9, was the cleaved form (p85), a substrate on which caspase-3 acts (Figure 3A, lane 8).

### Induction of Fas greatly enhanced Apaf-1- and caspase-9-mediated apoptotic cell death in U251** cells

**Figure 4:**
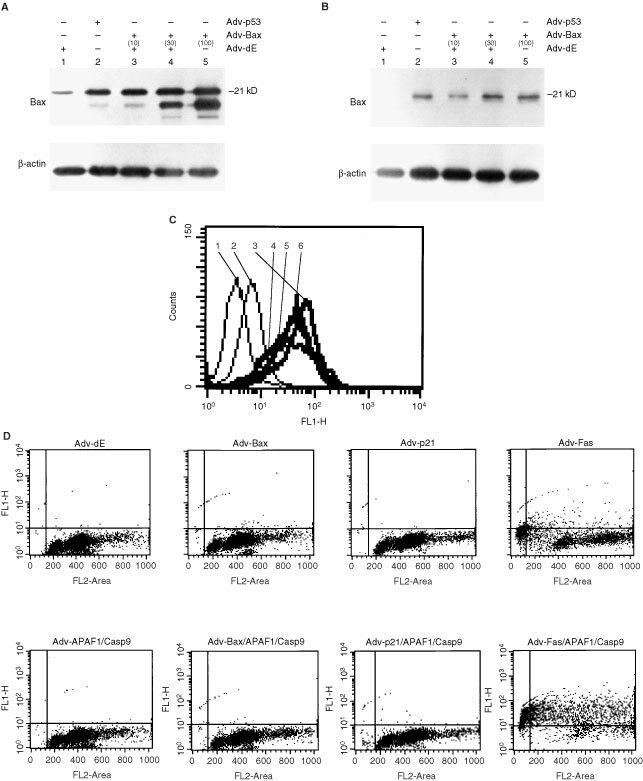
(**A**) Immunoblot analysis of Bax and β-actin protein extracted from U251 cells 48 h after being infected with Adv-p53, Adv-Bax and/or Adv-dE at various MOIs. The MOI of each Adv used to infect U251 cells is noted in parentheses. The total MOI was kept constant by supplementing with Adv-dE. Lane 1, U251 cells infected with Adv-dE (150); Lane 2, U251 cells co-infected with Adv-p53 (100) and Adv-dE (50); Lane 3, U251 cells co-infected with Adv-Bax (10), Adv-Cre (5) and Adv-dE (135); Lane 4, U251 cells co-infected with Adv-Bax (30), Adv-Cre (15) and Adv-dE (105); Lane 5, U251 cells co-infected with Adv-Bax (100) and Adv-Cre (50). (**B**) Immunoblot analysis of p21 and β-actin protein extracted from U251 cells 48 h after being infected with Adv-p53, Adv-p21 and/or Adv-dE at various MOIs. The MOI of each Adv used to infect U251 cells is noted in parentheses. The total MOI was kept constant by supplementing with Adv-dE. Lane 1, U251 cells infected with Adv-dE (100); Lane 2, U251 cells infected with Adv-p53 (100); Lane 3, U251 cells co-infected with Adv-p21 (10) and Adv-dE (90); Lane 4, U251 cells co-infected with Adv-p21 (30) and Adv-dE (70); Lane 5, U251 cells infected with Adv-p21 (100). (**C**) Expression of Fas on the surface of U251 cells as measured by FACS after being infected with Adv-p53, Adv-Fas or Adv-dE, or co-infected with various combinations of these Advs. The total MOI was kept constant by supplementing with Adv-dE. The data are presented as the log peak fluorescence intensity of U251 cells infected with the indicated adenovirus(es) and stained with the indicated antibody: (1) isotype-matched control 2 days after being infected with Adv-dE (MOI 150); (2) anti-Fas antibody 2 days after being infected with Adv-dE (MOI 150); (3) anti-Fas antibody 2 days after being co-infected with Adv-p53 (MOI 100) and Adv-dE (MOI 50); (4) anti-Fas antibody 2 days after being co-infected with Adv-Fas (MOI 10), Adv-Cre (MOI 5) and Adv-dE (MOI 135); (5) anti-Fas antibody 2 days after being co-infected with Adv-Fas (MOI 30), Adv-Cre (MOI 15) and Adv-dE (MOI 105); and (6) anti-Fas antibody 2 days after being co-infected with Adv-Fas (MOI 100) and Adv-Cre (MOI 50). (**D**) DNA fragmentation of U251 cells infected with Adv-Bax (MOI 10), Adv-p21 (MOI 30), Adv-Fas (MOI 100), Adv-APAF1 (MOI 100), Adv-Casp9 (MOI 100), Adv-dE, or co-infected with various combinations of these Advs. The total MOI was kept constant by supplementing with Adv-dE.

It has been reported that the apoptosis-related gene involved in p53-mediated apoptosis may be Bax (Miyashita and Reed, 1995) or Fas (Owen-Schaub *et al*, 1995). The p21/WAF1 gene is under the transcriptional control of p53 (el-Deiry *et al*, 1993). To evaluate through which gene p53 activates the Apaf-1 and caspase-9 genes, we co-infected the Adv for Bax (Adv-Bax), Adv for p21 (Adv-p21) or Adv for Fas (Adv-Fas) along with Adv-APAF1 and Adv-Casp9, and analyzed the degree of apoptosis. First, we determined the MOI of Adv-Bax, Adv-p21 or Adv-Fas that induces a similar level of Bax, p21 or Fas protein expression, respectively, as that after infection of Adv-p53 at an MOI of 100. Infection of Adv-Bax at an MOI of 10 ( Figure 4A

, lane 3) induced a similar level of Bax protein expression as that after infection of Adv-p53 at an MOI of 100 (Figure 4A, lane 2). Infection of Adv-p21 at an MOI of 30 (Figure 4B, lane 4) induced a similar level of p21 protein expression as that after infection of Adv-p53 at an MOI of 100 (Figure 4B, lane 2). Infection of Adv-Fas at an MOI of 100 (Figure 4C, No. 6) induced a similar level of Fas protein expression as that after infection of Adv-p53 at an MOI of 100 (Figure 4C, No. 3). Therefore, we infected Adv-Bax (MOI 10), Adv-p21 (MOI 30) or Adv-Fas (MOI 100) along with Adv-APAF1 and Adv-Casp9, and assessed the degree of apoptosis. The percentage of cells with fragmented DNA induced by co-infection of Adv-Bax, Adv-APAF1 and Adv-Casp9 in U251 cells was only 0.6% (Figure 4D, lower second left panel). Similarly, the percentage of cells with fragmented DNA in U251 cells co-infected with Adv-p21, Adv-APAF1 and Adv-Casp9, was only 0.5% (Figure 4D, lower second right panel). In contrast, U251 cells co-infected with Adv-Fas, Adv-APAF1 and Adv-Casp9 showed a drastically increased degree of apoptosis as high as 79% (Figure 4D, lower right panel), in comparison with that in U251 cells infected with Adv-Fas alone (10%) (Figure 4D, upper right panel). These results indicate that the upregulation of Fas by p53 might play an important role in the apoptosis induced by co-infection of Adv-p53, Adv-APAF1 and Adv-Casp9.

## DISCUSSION

In this study, we found that co-induction of Apaf-1 and caspase-9 greatly enhanced p53-mediated apoptotic cell death in comparison with that induced by the single induction of Apaf-1 or caspase-9. Caspase-9 associates with Apaf-1, and oligomerization of this complex in the presence of cytochrome *c* and dATP initiates the postmitochondrial-mediated caspase cascade by proteolytic activation of effector caspases, resulting in apoptosis (Li *et al*, 1997; Zou *et al*, 1997; Srinivasula *et al*, 1998). Indeed, electron microscopic analysis revealed damage of the mitochondria as early as 1 day after co-infection of Adv-p53, Adv-APAF1, and Adv-Casp9 in glioma cells (Figure 2D), suggesting that damage of the mitochondria preceded apoptotic cell death. How did p53 activate Apaf-1 and caspase-9? Several genes including Bax, p21 and Fas, have been reported to be upregulated by induction of p53 (Waga *et al*, 1994; Miyashita and Reed, 1995; Owen-Schaub *et al*, 1995). We transduced a similar amount of Bax, p21 or Fas protein into U251 cells via Adv vectors, as the level of the respective protein induced by p53. Transduction of Fas augmented Apaf-1- and caspase-9-mediated apoptosis (Figure 4D). Different genes such as Bax might be involved in p53-mediated activation of Apaf-1 and caspase-9 when p53 is transduced at a higher level. However, Fas played a critical role in activating Apaf-1 and caspase-9 at a relatively low level of transduction of p53 gene that did not induce drastic apoptosis. Indeed, in U251 and U-373MG cells, Adv-Fas-induced apoptosis was blocked by transduction of CrmA, inhibitor of caspase-8, which eventually activated downstream caspases by direct cleaving or inducing cytochrome *c* release from the mitochondria, resulting in the formation of Apaf-1/cytochrome *c*/caspase-9 complex (data not shown) (el-Deiry *et al*, 1993; Muzio *et al*, 1996; Zou *et al*, 1997; Budihardjo *et al*, 1999; Shinoura *et al*, 1999b; Slee *et al*, 1999). However, because Apaf-1, caspase-9 and cytochrome *c* form apoptosome in the presence of dATP, which activates the postmitochondrial-mediated caspase cascade, resulting in apoptosis, another factor needed for the enhancement of p53-inducing apoptosis by co-induction of Apaf-1 and caspase-9 might be several mitochondrial proteins that are induced by p53 as the direct-targets, such as Noxa and Puma (Oda *et al*, 2000; Nakano and Wousden, 2001). Further investigations are needed to decide the factors for the enhancement of p53-inducing apoptosis by co-induction of Apaf-1 and caspase-9.

Since mutation of the p53 gene is the most frequently-detected abnormality in human tumours, gene therapy specifically targeting tumour cells with mutated p53 has been performed. Preclinical and clinical (Phase I) studies have shown that restoring p53 function induces apoptosis and regression of tumours, suggesting that this therapy is feasible and safe (Roth *et al*, 1996). Therefore, it would be important to be able to predict the therapeutic effect of p53 gene therapy in a patient beforehand by analyzing the status of p53 or expression levels of p53-related genes (Fulci *et al*, 1998). Among gliomas, p53 gene therapy may be more effective on gliomas that express high levels of both Apaf-1 and caspase-9. In U251 and U-373MG cells, the endogenous level of Apaf-1 expression was relatively high, whereas the endogenous level of caspase-9 expression was relatively low (Figure 1). An imbalance in the levels of Apaf-1 and caspase-9 expression might render these gliomas resistant to p53 gene therapy. The therapeutic effect of p53 gene therapy has been shown to be augmented not only by chemotherapy (Fujiwara *et al*, 1994) or radiotherapy (Badie *et al*, 1999), but also by the induction of several genes including Fas (Rakkar *et al*, 1999), Fas ligand (Shinoura *et al*, 2000c) or p33^ING1^ (Shinoura *et al*, 1999c). Co-induction of Apaf-1 and caspase-9 enhanced the degree of p53-mediated apoptosis, suggesting that co-induction of p53, Apaf-1 and caspase-9 can be used as a modality for the gene therapy of cancers. Further investigation is required to apply this strategy to the gene therapy of gliomas.
